# Accessing and engaging women from socio-economically disadvantaged areas: a participatory approach to the design of a public health intervention for delivery in a Bingo club

**DOI:** 10.1186/s12889-016-3013-1

**Published:** 2016-04-18

**Authors:** Josie M. M. Evans, Gemma Ryde, Ruth Jepson, Cindy Gray, Ashley Shepherd, Dionne Mackison, Aileen V. Ireland, Marion E. T. McMurdo, Brian Williams

**Affiliations:** School of Health Sciences, University of Stirling, Stirling, FK9 4LA Scotland, UK; Scottish Collaboration for Public Health Research and Policy, 20 West Richmond St, EH8 9DX Edinburgh, UK; Institute of Health and Wellbeing, College of Social Sciences, University of Glasgow, Glasgow, G12 8RS UK; NHS Health Scotland, Edinburgh, UK; Medical Research Institute, University of Dundee, Ninewells Hospital, Dundee, DD1 9SY UK

**Keywords:** Physical activity, Intervention, Participative approach, Novel setting

## Abstract

**Background:**

Our aim was to use participatory methods to investigate the feasibility and acceptability of using Bingo clubs for the design and delivery of an evidence-based physical activity and/or healthy eating intervention to socio-economically disadvantaged women. This paper describes the participatory process that has resulted in a physical activity intervention for women aged >55 years, ready for pilot-testing in a Bingo club setting.

**Methods:**

Studies using different quantitative and qualitative approaches were conducted among customers and staff of a Bingo club in a city of 85,000 inhabitants in central Scotland. These were designed to take the views of different stakeholders into account, with a view to enhancing uptake, engagement and effectiveness with any proposed intervention.

**Results:**

Sixteen relevant studies were identified in a literature review that generated ideas for intervention components. A questionnaire completed by 151 women in the Bingo club showed that almost half (47 %) aged >55 years were not meeting physical activity guidelines; evidence backed up by accelerometer data from 29 women. Discussions in six focus groups attended by 27 club members revealed different but overlapping motivations for attending the Bingo club (social benefits) and playing Bingo (cognitive benefits). There was some scepticism as to whether the Bingo club was an appropriate setting for an intervention, and a dietary intervention was not favoured. It was clear that any planned intervention needed to utilise the social motivation and habitual nature of attendance at the Bingo club, without taking women away from Bingo games. These results were taken forward to a 5-h long participative workshop with 27 stakeholders (including 19 Bingo players). Intervention design (form and content) was then finalised during two round table research team meetings.

**Conclusions:**

It was possible to access and engage with women living in areas of socio-economic disadvantage through a Bingo club setting. A physical activity intervention for women >55 years is realistic for recruitment, will address the needs of potential recipients in the Bingo club, appears to be feasible and acceptable to club members and staff, and has been designed with their input. A pilot study is underway, investigating recruitment, retention and feasibility of delivery.

## Background

Poor diet and lack of physical activity are major public health problems [1] and are associated with adverse socio-economic position in Scotland [2–4]. Women from socio-economically disadvantaged backgrounds are at increased risk of a range of associated health problems, including type 2 diabetes, cardiovascular disease and cancer. Despite this, many women from deprived areas are excluded from settings used for the delivery of targeted public health interventions, for example schools and workplaces. Consequently, there is a need to explore how to develop novel interventions with sufficient reach to meaningfully access and engage this group.

Both social environment and social cohesion in particular, have been highlighted as predictors of uptake and engagement with interventions [5]. A potentially powerful approach is to use pre-existing social groups and networks to deliver health behaviour interventions. There has, for example, been a fairly strong tradition of using churches for health promotion in the United States [6], but such settings have been less commonly used in the UK. There is then the further challenge of maintaining any behaviour change that results from delivery of a relatively short intervention [7].

Uptake and engagement with public health interventions, and consequently their effectiveness, can also be compromised because insufficient attention is paid to the culture, values and concerns of the intended recipients. The importance of engaging with local communities and involving multiple stakeholders in the research process is therefore increasingly recognized [8]. There is growing interest in a community-based participatory research (CBPR) approach to the development, delivery and evaluation of health interventions; involving community members as equal partners in all stages of the research process. While a full CBPR process may not be appropriate or feasible in all situations [9], it has been used in the delivery of multi-faceted interventions to marginalised groups [10, 11], in exploratory studies [12, 13] and in intervention design [13].

With this in mind, we identified a novel setting for the design of a health intervention for socio-economically disadvantaged women in the UK, and used elements of a CBPR approach to investigate its potential. Bingo clubs run games sessions at different times every day of the week, and are accessed by hundreds of people, with the socio-demographic group of interest well-represented [14].

The aims of this study were to investigate the feasibility and acceptability of using a commercial Bingo club for delivery of an evidence-based health intervention to socio-economically disadvantaged women, and to use participatory methods in its development.

## Methods

This study used elements of a CBPR approach [8] and was carried out in a commercial Bingo club in a city of around 85,000 inhabitants in Central Scotland. For the purposes of CBPR, the community was defined as Bingo club stakeholders: the manager, members of staff and club members. To represent this ‘community’ at research team meetings, two club members and one staff representative from the Bingo club were invited to join the research team and were given shopping vouchers for their time. At least one ‘lay member’ was present at every research team meeting held in the club and they also attended focus groups and workshops. There were two phases to the project. The purpose of Phase 1 was to explore the wider context by reviewing the scientific literature to identify health interventions delivered in similar settings and circumstances, and to assess the socio-demographic characteristics and health behaviours of women attending the Bingo club. In Phase 2, data surrounding barriers and facilitators to behaviour change among Bingo club members were generated to inform intervention type and content, which was then designed with input from them.

### Phase 1

#### Literature review

A literature review was conducted to identify group-based physical activity and healthy eating interventions delivered to community-recruited women from socio-economically disadvantaged backgrounds. Electronic searches were conducted using Pub Med, Web of Science, SPORTDiscus, Medline and PsycInfos in July 2013 using groups of thesaurus terms and free terms. Search terms for ‘women’ were used in AND-combinations with terms for ‘socio-economic status’, ‘intervention’, and ‘healthy behaviour’ (physical activity and food) with no limit on date range applied. Additional articles were identified by manually checking reference lists of reviews identified in the search. For grey literature, individuals in key public health organisations were contacted, including National Health Service (NHS) Health Scotland Health Promotion (national and regional), NHS central librarian, Community Food and Health Scotland, and the Physical Activity and Health Alliance.

The titles and abstracts of all published papers were examined for relevance. Studies were included if they were available in English and described a group intervention that addressed physical activity and/or healthy food in an adult community-recruited population of mainly (>85 %) women. Clinical populations were excluded, as were those with long term health conditions, those recruited because of a specific illness, and interventions conducted in primary care settings. Studies specifically targeting adults from low socio-economic groups were included, regardless of how this was defined. The intervention had to have a group-based component with repeated group and/or social contact with other participants.

The interventions identified were classified as targeting physical activity, diet, or both; and those that were effective were examined further to identify the intervention content, and any behavioural change strategies that were employed.

### Questionnaire and accelerometers

In order to gather information about Bingo players’ dietary and physical activity behaviours to help inform the content of any intervention, a questionnaire addressing these behaviours was piloted among 24 club members, then administered to a convenience sample of Bingo players as they came in for Bingo sessions. The final questionnaire incorporated a questionnaire from the Active Australia Survey [15], and the Dietary Intervention in Primary Care (DINE) [16] measure. Active Australia uses a self-report physical activity questionnaire that asks respondents to recall types and intensity of physical activity carried out over the previous week. The DINE questionnaire measures dietary intake of fibre and fat, with additional questions on fruit and vegetable intake. To supplement the self-reported physical activity data, which can over-estimate physical activity levels, objectively-measured accelerometer data were collected over a one-week period among a sub-sample of questionnaire respondents. Data were then sorted into activity categories using established vector magnitude count-based thresholds as follows: sedentary (<150), light (151 to 2689) and moderate to vigorous activity (>2690) [17].

### Phase 2

#### Focus groups

We used formative qualitative research methods to explore the range of stakeholder views on promotion of healthy lifestyles and what sort of interventions might be suitable for delivery in a Bingo club. Participants were recruited face-to-face at a manned stall in the Bingo club during one week in April 2013. They were asked if they would be interested in taking part in a ‘health project at the club’ where they would discuss ways in which they could make their lives healthier. Those interested were then invited to attend a focus group which lasted around an hour and was held at the club before or after Bingo sessions, and they received £15 of shopping vouchers for their attendance. Focus groups were considered an appropriate method of qualitative data collection given the importance of pre-existing social networks. Six focus groups were held; they were facilitated by GR with another co-applicant (and sometimes a lay member) always present for note taking. The focus groups were structured around six key questions (Table 1). However, the participants were allowed time to discuss other issues freely as they arose. They found it particularly difficult to come up with their own ideas, unprompted, for ways in which the Bingo club could be used to promote healthier lifestyles. Therefore GR initially used examples of similar interventions identified from the literature for discussion. However, as the focus groups progressed, she was able to present ideas for discussion that had arisen in previous focus groups.

**Table 1:** Protocol for focus groups

Protocol question
What are the benefits you gain from playing bingo?
What do you think of when I say ‘healthy lifestyles’?
Can you give me some examples of what you or others you know have done to have a healthier lifestyle?
Which aspect of a healthy lifestyle is most important to you?
Think about your own and others experiences, what do you think makes having a healthy lifestyle difficult and what can help to make it easier?
Can you think of any ways in which the Bingo club could be used to help promote healthy lifestyles?

The focus groups were digitally recorded, transcribed and analysed by GR and other co-applicants (RJ, JE, GR, AI) using a thematic framework.

### Key stakeholder interviews

Informal conversations took place between members of the research team and the Bingo club manager or one of his two assistant managers on several occasions, and these were written up electronically shortly afterwards. At their request, these informal conversations replaced the formal audio-recorded interviews that had originally been planned.

### Participatory workshop

In order to involve Bingo players and staff in the specific design of the intervention and to tailor it to the Bingo context, a 5-h-long participatory workshop was held in February 2014 at a local hotel to elicit their views. Invitations to the workshop were sent to 36 Bingo club members. These included focus group participants, and women who had initially expressed an interest in the study and for whom we had postal addresses. The event was led by the research team and included a short presentation on the project so far, videos of possible activities (identified from the literature review and focus groups), a chair-based activity session led by the local Council provider, small group discussions of strategies to attract/retain participants, and participant feedback and indication of preferences. Detailed note-taking was undertaken by research team members during group discussions. We used a strategy to elicit activity preferences whereby women placed coloured dots on possible ideas for an intervention on a large flip chart, providing a very visual representation of the results. This ensured that women with stronger views or who were more articulate did not have undue influence. The workshop was not audio-recorded (at some participants’ request) but reflective notes were written up by all researchers at the close of the workshop, which were then synthesised. Participants were also asked to complete a short activity preference questionnaire to guide discussion.

### Round table meetings

Two round table meetings with members of the research team were held in April and May 2014 to finalise the intervention. During the first meeting, summary evidence from each of the phases was presented to the group. This included evidence from the literature review on techniques used in other effective interventions, results from the questionnaire, and focus group and workshop-generated data. This generated ideas for intervention components. In the second meeting, these components were specified in greater detail.

### Ethics

This study was approved by the Research Ethics Committee of the School of Health Sciences, University of Stirling.

## Results

### Phase 1

#### Literature review

From an original list of 2,667 academic papers, 120 full texts were reviewed and 16 relevant studies (covering 14 interventions) were identified that described and/or evaluated a physical activity or healthy eating intervention specifically targeted at community-recruited women from socio-economically disadvantaged backgrounds [18–33]. Ten interventions addressed exercise or physical activity [18–20, 22, 24, 27–32], nine targeted diet [18, 21–26, 30, 31, 33] and five targeted both [18, 22, 24, 30, 31]. Included papers were of variable quality (some had no control groups or validated measures) making assessment of effectiveness difficult. Intervention components varied, with ten including some form of education.

When the 15 physical activity interventions were examined in more detail, six of the studies measured and/or reported physical activity-related outcomes [18–20, 24, 27, 29, 31]. There was evidence for effectiveness at changing physical activity behaviour for three interventions [19, 20, 24, 27], and one for increasing physical activity knowledge [18]. Three of these four effective interventions included an educational component and drew upon social cognitive theory [18], the trans-theoretical model [19, 20] and trans-operant theory [24], respectively. Using Abraham and Michie’s taxonomy of behaviour change techniques [34], those strategies that were used in the four effective interventions were identified (Table 2).

**Table 2:** Behavioural change techniques used in four effective physical activity interventions [34]

Technique	How many of the 4 effective interventions used this technique
Provide general information linking behaviour to health	1
Provide information on consequences	2
Prompt intention formation	2
Prompt barrier identification	3
Provide instructions	2
Model / demonstrate the behaviour	2
Provide rewards contingent on behaviour	2
Prompt practice	1
Use follow-up prompts	2
Provide opportunities for social comparison	2
Plan social support / social change	3
Time management	2

### Questionnaire/ accelerometer data

There were 162 questionnaires returned. A small number of men (11) completed the questionnaires, but their results were excluded from further analysis. Almost all (97 %) female respondents were white Scottish. Mean age was 57 ± 17.7 years (range 18–91 years). The majority (57 %) lived in the two quintiles of highest deprivation. This was assessed using a post code measure of material deprivation known as the Scottish Index of Multiple Deprivation (SIMD) [35]. 69 % said they were in good, very good or excellent health, despite 53 reporting that they had a condition that affected their health.

The mean number of fruit and vegetables consumed daily by 144 questionnaire respondents was 4.7, and only 19 % reported a high dietary fat intake. Of those who completed the physical activity questions (*n* = 149), 37 % were found not to be meeting the 2012 UK physical activity guidelines (defined as 150 min/week [36]), with this proportion higher among respondents >55 years (46 %), than those <55 years (23 %). In total, 29 women agreed to wear accelerometers for one week. Of these, the majority were aged >55 years (*n* = 20), with the remaining being aged <55 years (*n* = 9). Mean daily physical activity minutes were 22 and 53 in the older and younger women respectively. The women aged >55 years spent 61 % of their time sedentary, compared to 52 % sedentary time among younger women.

### Phase 2

During the recruitment week, 91 women expressed an interest in taking part in the study. Six focus groups were then conducted with 27 women from a range of backgrounds. As Table 3 shows, it was difficult to recruit younger women (18–55 years) to the focus groups, despite additional groups being organised specifically at times/locations to attract them. A higher proportion of women living in areas of deprivation than those from less deprived areas, did not turn up to scheduled focus groups, but the higher proportion of women from deprived backgrounds who initially expressed an interest (reflecting the higher proportion within the study population) meant that they were still well-represented at focus groups.

**Table 3:** Attendance at focus groups

Age (years)	Expression of interest in study (EOI) (*n* = 91)	Focus group attendance (*n* = 27)	% attendance of EOI	SIMD [35]	Expression of interest in study (EOI) (*n* = 91)	Focus group attendance (*n* = 27)	% attendance of EOI
<45	12	1	8 %	1	21	5	24 %
46-55	28	1	4 %	2	34	7	21 %
56-65	17	9	53 %	3	20	7	35 %
66-75	17	8	47 %	4	12	6	50 %
>75	17	8	47 %	5	2	2	100 %

Table 4 presents findings from the focus groups relevant to intervention design. For example, there were different but overlapping motivations for attending the Bingo club and playing the game of Bingo. A key motivation was social; many women also felt that taking part in Bingo had cognitive benefits. In terms of perceptions of healthy lifestyle, healthy eating was considered to be more important than physical activity. Many women already considered themselves to be busy and ‘active’, although the definition of ‘active’ differed considerably between them, as they had varying levels of mobility. In general, there was scepticism as to whether the Bingo club was an appropriate setting for a health intervention, although some women remained open-minded about this. Many women came to Bingo to relax and for them physical activity contradicted this perceived benefit. A strong view shared by players and staff was that any intervention should not take women away from their primary reason for being there.

**Table 4:** Summary of focus group findings, with illustrative quotes

Focus group findings	Illustrative quotes
There are different but overlapping motivations for attending the Bingo club and playing the game of Bingo. Key motivations among many women for attendance are socialising and enjoyment, while many feel that taking part in Bingo has cognitive benefits for them.	“It keeps the wee grey cells going” [laughs]. *Participant 1 focus group 4* “It’s a great thing for older people just keeps you as you say alert, that’s it. Plus the company”. *Participant 3 focus group 1* “Everyone says ‘hello’ and ‘how are you’ and that’s about it, basically why we come isn’t it”. *Participant 4 focus group 6* “To me it’s a day out on a Friday, this is my day out and I thoroughly enjoy it”. *Participant 3 focus group 4* “I love it. Well I enjoy it” [agreement]. *Participant 3 focus group 5*
In terms of what women perceive constitutes a healthy lifestyle, healthy eating was more frequently mentioned and discussed than physical activity. Many women already consider themselves to be busy and active, and eat healthily.	“Well I’ve got two Scottish Deerhounds …erm… that says it all I think and I’ve got to walk them every day. Erm… and it’s miles you walk it’s, my Deerhound is big but I do walk miles every day …erm… and that’s a huge part of your life.” *Participant 2 focus group 2*. “I can eat anything but I like home made things I like to make everything from scratch, I don’t buy ready meals, I don’t like them, I have to cook like, yesterday I had my daughter coming for her dinner and I had, I made homemade soup, then I made mince with butter beans in it and…”. *Participant 4 focus group 4* “I think that is how half of us are living as long for the simple reason we had good food when we were younger and as I say once, well I’m left on my own now, and as I say you just make good with whatever you feel like but I mean we were never brought up to waste food and that and the amount of food that is wasted now is something terrible.” *Participant 4 focus group 5*
There is scepticism as to whether the Bingo club is an appropriate setting for a physical activity intervention, although some women remain open-minded about this. Many women come to Bingo to relax and for them physical activity contradicts this perceived benefit.	“And say you are doing this and you are doing that and you will get a lot of them will not do it but I think you would get a lot that would” Participant *2 focus group 1* “And some of them would maybe tell you to bugger off” [laughs]. *Participant 1 focus group 6* “It would be interesting to know what you do do but I don’t think it would interest a lot of people that come here to be quite honest as long as they are getting a game of bingo and going out for a cigarette and getting a pint at the bar…”. *Participant 2 focus group 6* “Anyway they want to play their mini bingo before the bingo starts so where would you have time to do exercise”. *Participant 1 focus group 3* “Because folk go to the bingo and they just want to go to the bingo and no strings attached”. *Participant 3 focus group 3*
Any planned intervention needs to exploit the social motivation and habitual nature of attendance at the Bingo club.	“Creatures of habit” [agreement and laughter]. *Participant 3 focus group 3* “But a lot of people, well that’s what we all do but there’s a lot of people have set days for doing things.” *Participant 2 focus group 6* “And a lot of them are all set in their ways and everything…”. *Participant 3 focus group 6* “Uhuh I mean that’s like a lot of folk that have got busy life and they go to the bingo for a set day and have a blether with folk…”. *Participant 4 focus group 5*
It is important that any intervention planned in the Bingo club setting does not take women away from their primary reason for being there (Bingo) and the core business of the club (to make money). This very strong view is shared by players and staff.	“They are wanting to make money.” *Participant 1 focus group 3* “But can you imagine the management not putting on mini bingo that’s where they get their money. *Participant 1 focus group 6*

### Participatory workshop

The workshop was attended by 27 people. There were 19 Bingo players (three of whom had not attended an earlier focus group), two of the lay research team members (one player, one member of staff), four other members of the research team, and two people from the local Council. Most of the Bingo players were aged >55 years, with only two younger participants.

The results (Table 5) clearly indicated that the participants did not support any form of walking intervention (identified as a possible intervention component from the literature). They gave a strong steer towards group-based, led, manageable and short activities that included music, that were not seen as traditional exercise and did not take place outdoors. They expressed dislike for traditional education sessions and were not supportive of ‘buddy systems’, also identified from the literature. Women were able to contribute useful ideas for Bingo-related incentives for attendance.

**Table 5:** Workshop-generated data

Activity Preference Questionnaire^a^				Workshop “voting”^b^			
	Agree	No pref.	Dis-agree	Activity type		Strategies	
Format						Incentives	
Involve competition	12	2	4	Aerobics	0	Payment of £1 per session to win kitty or fund trips to other clubs	27
Require skill and practice	9	7	3	Boccia	2	Attendance loyalty card	10
Have a set routine or format	9	10	1	Chair based exercise	24	Free bingo for achieving targets	16
Involve little or no cost	11	9	0	Dancing	2	Competitions	0
Involve supervision (eg, from a leader)	15	2	3	Gentle exercising	1	Certificate for attendance or goals	3
Are team based	8	10	2	Healthy living classes	4	Knowledge and information	
Are done at a fixed time (eg, scheduled sessions)	11	6	3	Jazzercise	3	Group based information session	4
Are not just about exercise	12	5	2	Line dancing	13	Leaflets	0
Are vigorous (make me breathe harder)	10	3	7	Pilates	2	Notice board	8
Are done in a small group (eg, classes at a club or centre)	15	5	1	Scottish country dancing	0	Social support	
Social setting				Step Aerobics	2	Role models	0
I can do on my own	10	7	3	Stretching	7	Text message reminders	1
Are done with others	13	5	1	Tai Chi	3	Online forum	0
Are done with people around my age	14	5	1	Tea dancing	1	Website	0
Are done with people of my own sex	6	12	2	Walking indoors	7	Telephone support	0
Are done with people at my level of ability	10	8	1	Walking for health outdoors	2	Buddy system	3
Location				Walking with poles	3	Planning, recording and measuring achievement	
Can be done close to home	17	2	1	Weights	0	Goal setting, monitoring and feedback	0
Are done outdoors	6	11	3	Wi Fit	1	Optional health measurements	0
Are done at home	11	9	0	Yoga	0	Step counters	4
				Zumba	3		

^a^19 respondents

^b^All players at workshop given 10 coloured dots each to use/not use as they wished

### Round table meetings

The two round table meetings were each attended by five and six members of the research team respectively. Although the lay members were invited to the round table research meetings, none were able to attend. One was ill and another had been finding it difficult to devote time to the project. It is possible that the venue (University campus) may have deterred the third from attending. However, it would have been difficult to hold these longer and more involved meetings at the Bingo club, where there are distractions from noise and other activity even outwith Bingo sessions.

In the first meeting, the intervention components were identified. Bringing together all the evidence and after a lengthy discussion, a consensus emerged that the intervention would consist of physical activity sessions, the specific nature of which would be chosen by potential participants themselves in a preliminary workshop (as part of the intervention), but which would be delivered indoors in the Bingo club. Participants would devise their own incentives for attendance. There would be time for social interaction and the sessions would also be used as a platform to deliver simple key health messages, for which instructors would be trained. The messages would not be delivered overtly, but where possible would be incorporated naturally into participant discussions.

In the second meeting, these components were specified in more detail. It was agreed that 20–30 min sessions, with an equal length of time for socialising with the instructor present, would be appropriate. Much of this meeting was then devoted to designing a week by week schedule of key messages to be subtly delivered to participants.

## Discussion

As a primary output of this project, a physical activity intervention tailored for women aged >55 years, has been designed for delivery in a setting where women living in areas of socio-economic disadvantage are well-represented. Our work has shown that the Bingo club is a suitable setting for reaching women living in socio-economically deprived areas. Nearly half (44 %) of the focus group participants lived in areas of SIMD1/2, as did 57 % of all research participants.

There were several factors underpinning the decision to target older women in this setting with a physical activity intervention. The Bingo club manager estimated that some 75-80 % of around 2,500 club members were over the age of 45 years, and it was thought probable, even before the research commenced, that we might be working with an older group of women. While some younger women did express an interest in the study, only two women <55 years attended focus groups, despite organising these at times and locations that would be convenient for them. A pragmatic decision was therefore made to tailor the proposed intervention to women aged >55 years who were less likely to be working during the day.

In focus group discussions, many women (particularly older women) reported reasonable dietary habits, and aspects of this were confirmed by the questionnaire which demonstrated satisfactory levels of fruit and vegetable consumption. In contrast, although they did not self-identify as needing to increase physical activity, around one-third of respondents were not meeting physical activity guidelines. This proportion was even higher (47 %) among women >55 years. It is difficult to know how representative the questionnaire respondents were of the wider Bingo club membership, as this was a convenience sample of an unknown total number of players (including 11 men) coming in and out of selected (but diverse) Bingo club sessions during a one-week period. However, they appeared to be broadly representative of club members in terms of socio-demographic characteristics.

During Phase 2, the possibility of designing a healthy eating intervention had been retained. However, discussions with the Bingo club managers had indicated that there would be very significant challenges associated with trying to change menus in the club’s catering outlet. Given that there was a noted lack of enthusiasm for educational sessions among women at the workshop (possibly the only viable way to deliver a healthy eating intervention in this setting), the decision was made to target physical activity behaviours.

The content and design of the intervention was finalised during the participatory workshop . We drew upon and synthesised multiple sources of information in this phase to ensure that the intervention would be acceptable to its intended recipients, but also underpinned by evidence. Initial ideas for intervention components were generated from focus groups and from the literature. While it was difficult for focus group participants to come up with ideas with limited knowledge of physical activity interventions and for a behaviour for which they did not self-identify a need, the focus groups did give a clear direction in terms of motivations and facilitators for women attending the Bingo club itself, and the final intervention reflects these. The views of women at the participatory workshop then very much shaped the components that were included in the final intervention. We drew upon theoretical models to inform the content of the educational messages and to incorporate behavioural strategies that had been used in other effective interventions where appropriate.

The project has shown that it is possible to access and engage with women living in areas of socio-economic disadvantage through a Bingo club setting for discussions around health, and to develop a health intervention. Use of the CBPR approach involved canvassing the views of all Bingo stakeholders. Support from the Bingo club itself was crucial both in terms of access to the premises itself and visible endorsement of the project. Involving lay members on the research team also provided an insider’s perspective of what was possible at the club, including the commercial viewpoint from the staff member. The lay members’ involvement with the project also created trust with other players and increased the project’s credibility in the club. However, this involvement was not problem-free. In particular, there were sensitivities during discussions about Bingo clubs as a setting to recruit women from areas of socio-economic disadvantage, as players do not necessarily self-identify as being disadvantaged. But while having lay members on the research team assisted with the visibility and endorsement of the project, other research team members also put considerable time investment into playing Bingo themselves, and becoming ‘known’ at the club.

While there was support for the proposed physical activity intervention at the Bingo club among workshop participants, whether this support will be reflected in the wider club membership is uncertain. We initially hypothesized that recruitment to and retention in any proposed intervention would be enhanced by taking a CBPR approach and working with a pre-existing social group; this is now being evaluated in a pilot study, as is feasibility of delivery.

## Conclusion

The most important lesson learned for public health intervention design in other novel settings is the importance of buy-in from all stakeholders to ensure feasibility. We have been successful in accessing and engaging with Bingo players in the design of a health intervention which will have considerable potential for reaching large numbers of women from areas of socio-economic disadvantage. A specific intervention (the Well!Bingo physical activity intervention) has been designed for women >55 years and is now undergoing feasibility testing in the Bingo club. It consists of five core components, each of which can be adapted to suit the requirements of individual clubs, comprising structured exercise sessions, intervention messages, a social component, Bingo-related attendance strategies and specific training of instructors. The crucial question now is whether the intervention is effective, and whether its effectiveness has been enhanced by the approach we have taken. If so, this will have very important implications for intervention design in other novel settings.

## Abbreviations

CBPR: community-based participatory research
DINE: Dietary Intervention in Primary Care
NHS: National Health Service
SIMD: Scottish Index of Multiple Deprivation
